# TRPM8 in the negative regulation of TNFα expression during cold stress

**DOI:** 10.1038/srep45155

**Published:** 2017-03-23

**Authors:** Xin-Pei Wang, Xuan Yu, Xiao-Jin Yan, Fan Lei, Yu-Shuang Chai, Jing-Fei Jiang, Zhi-Yi Yuan, Dong-Ming Xing, Li-Jun Du

**Affiliations:** 1 MOE Key Laboratory of Protein Sciences, Laboratory of Molecular Pharmacology and Pharmaceutical Sciences, School of Life Sciences, Tsinghua University, Beijing 100084, China; 2 School of Pharmacology and Pharmaceutical Sciences, Tsinghua University, Beijing 100084, China

## Abstract

Transient Receptor Potential Melastatin-8 (TRPM8) reportedly plays a fundamental role in a variety of processes including cold sensation, thermoregulation, pain transduction and tumorigenesis. However, the role of TRPM8 in inflammation under cold conditions is not well known. Since cooling allows the convergence of primary injury and injury-induced inflammation, we hypothesized that the mechanism of the protective effects of cooling might be related to TRPM8. We therefore investigated the involvement of TRPM8 activation in the regulation of inflammatory cytokines. The results showed that TRPM8 expression in the mouse hypothalamus was upregulated when the ambient temperature decreased; simultaneously, tumor necrosis factor-alpha (TNFα) was downregulated. The inhibitory effect of TRPM8 on TNFα was mediated by nuclear factor kappa B (NFκB). Specifically, cold stress stimulated the expression of TRPM8, which promoted the interaction of TRPM8 and NFκB, thereby suppressing NFκB nuclear localization. This suppression consequently led to the inhibition of TNFα gene transcription. The present data suggest a possible theoretical foundation for the anti-inflammatory role of TRPM8 activation, providing an experimental basis that could contribute to the advancement of cooling therapy for trauma patients.

Transient Receptor Potential Melastatin-8 (TRPM8) has been identified as a cold-activated non-selective cation channel expressed in a small population of peripheral sensory neurons^1^,^2^. TRPM8 is also widely expressed in non-temperature-sensing organs^3^,^4^,^5^,^6^,^7^. During the past few years, the involvement of TRPM8 in cold sensation has been well-documented^8^,^9^. TRPM8-deficient mice exhibited no preference for the optimum ambient temperature and impaired cold avoidance behavior^10^. Controlled cooling, accompanied by the activation of TRPM8, contributed to diminished neuropathic and visceral pain^11^,^12^ and attenuated the inflammatory reaction^13^ and nerve injury pain^14^. Meanwhile, tissue cooling or hypothermia has been widely used to suppress tissue damage resulting from trauma, ischemia and surgery^15^ and to inhibit inflammation^16^,^17^. These findings suggest that TRPM8 may possess an anti-inflammatory effect in certain circumstances.

Thus, we conducted *in vivo* and *in vitro* experiments to investigate the mechanism of the negative correlation between TRPM8 and TNFα. In this study, we confirmed that the expression of TRPM8 in mouse hypothalamic neurons is activated by cold stress, suggesting that the expression of cold-sensitive TRPM8 channels is also distributed along the central neural system. In addition, we observed that when the expression of TRPM8 in the mouse hypothalamus was upregulated in cold environments, TNFα expression was downregulated.

## Results

### Expression of cold-sensitive TRPM8 and TRPA1 in mouse brains

**Figure 1:**
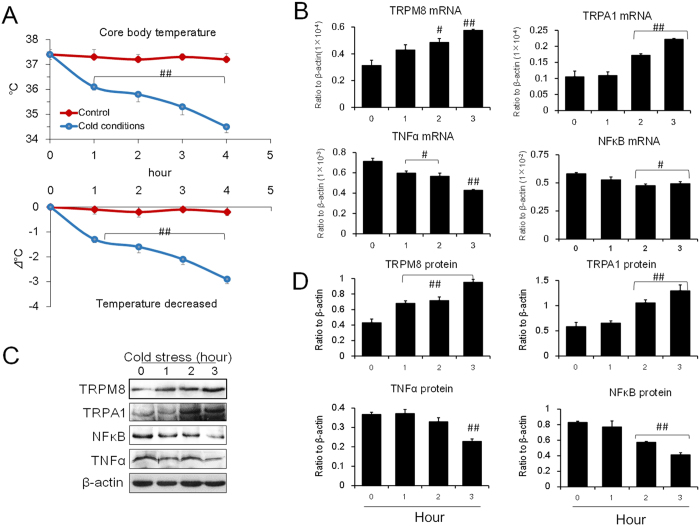
Alteration of core temperature and the expression levels of TRPM8, TRPA1, NFκB and TNFα in mouse brains under cold conditions. (**A**) Core body temperature under cold conditions (4 °C). (**B**) The mRNA expression levels of TRPM8, TRPA1, NFκB and TNFα. (**C,D**) The protein expression levels of TRPM8, TRPA1, NFκB and TNFα. Data are shown as the mean ± S.D. from 12 mice in each group. ^##^*v.s*. the control (zero hour), *P* < 0.01; ^#^*P* < 0.05.

To investigate the expression of TRPM8 in the central neuronal system of mice and its response to cold stress, mice were placed under cold conditions (4 °C) or normal conditions (25 °C). As shown in Fig. 1A, under prolonged cold exposure, the rectal temperature (core body temperature) of the mice decreased significantly. The temperature of mice in the test groups decreased by 3 °C compared to control groups after 3 hours in cold conditions (Fig. 1A). Interestingly, both the mRNA and protein expression levels of TRPM8 and TRPA1 in the mice hypothalamus were upregulated (Fig. 1B–D; Supplementary Fig. S8). At the same time, mRNA and protein expression levels of TNFα and NFκB were downregulated, suggesting a relationship between TRPM8 and TNFα.

### Expression levels of TRPM8 and TNFα *in vitro* under cold conditions

Since temperature perception and thermoregulation in mammals depends on both neural and humoral regulation, it is difficult to verify whether cold conditions influence the activation of thermo-sensitive transient receptor potentials (TRPs) and inhibit inflammatory cytokines. We therefore conducted *in vitro* experiments to evaluate the effects of cold conditioning using PC12 cells, a neural-like cell line^18^.

**Figure 2:**
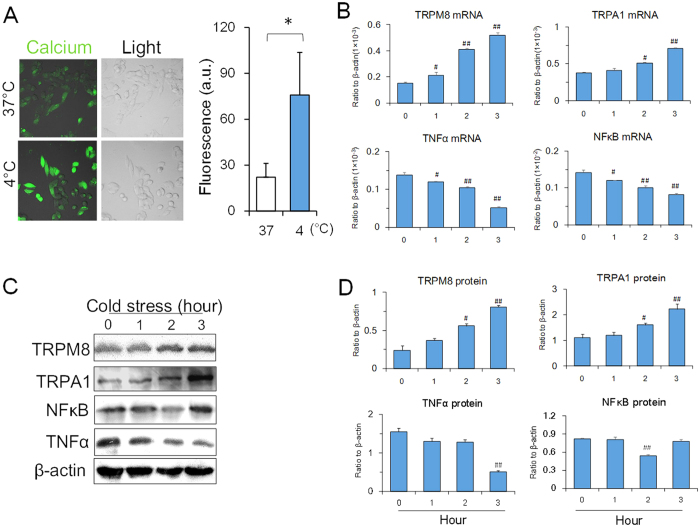
Expression of TRPM8, TRPA1, NFκB and TNFα in PC12 cells under cold conditions. (**A**) Intracellular Ca^2+^ in the cells under cold conditions (4 °C). The Ca^2+^ concentration in the cytoplasm at 4 °C is higher than that at 37 °C. (**B**) The mRNA expression levels of TRPM8, TRPA1, NFκB and TNFα. (**C,D**) The protein expression levels of TRPM8, TRPA1, NFκB and TNFα. Data are shown as the mean ± S.D. from three experiments. ^#^*P* < 0.05; ^##^*P* < 0.01, v.s. the control (zero time).

In Fig. 2A, intracellular calcium was higher under cold conditions than under normal conditions, suggesting that cold stress was able to stimulate calcium influx and activation. At the same time, mRNA and protein expression levels of TRPM8 and TRPA1 in the cytoplasm increased, while mRNA and protein expression levels of TNFα in the cytoplasm decreased (Fig. 2B–D; Supplementary Fig. S8). At different temperatures (4 °C and 20 °C), it was found that TRPM8 expression was more sensitive to the lower temperature (4 °C) than TRPA1 expression, and TNFα decreased significantly at 4 °C, suggesting that the expression of TRPM8 was more closely related to TNFα downregulation (Supplementary Fig. S1). Further, comparing with 4 °C, the upregulation of TRPM8 expression and downregulation of NFκB and TNFα were limited under 34.5 °C and 30 °C. In 34.5 °C (the core body temperature of mice during cold conditions), there was no significant change in the expressions of TRPM8, NFκB and TNFα compared to the control. In 30 °C, the expressions of TRPM8, NFκB and TNFα in the cells showed a tendency of up and down but without statistical significance. However, in 20 °C, the expressions of TRPM8, NFκB and TNFα in the cells showed a difference from the control distinctly, suggesting lower temperature could effectively stimulate the factors response and the response of TRPM8, NFκB and TNFα was in a temperature-dependent manner (Supplementary Fig. S2). However, when in the heat conditions (40 °C), the mRNA expressions of HSP70, NFκB and TNFα was activated in both mice and PC12 cells under heat conditions, while TRPM8 was inhibited by heat conditions (Supplementary Fig. S3). The results indicated that mechanism under heat condition was different from that in cold condition. In agreement with the *in vivo* experiments, cold stress is able to upregulate the mRNA and protein expression levels of TRPM8 and TRPA1 in PC12 cells, while downregulating the expression of TNFα and NFκB (Fig. 2B,D), suggesting that the lower temperatures might directly activate TRPM8 and TRPA1 expressions in cells.

### Cold-induced effects in Trpm8 knockdown cells

To further confirm the relationship between TRPM8 and TNFα, siRNA assays were employed. When *Trpm8* siRNA was introduced into the cells, the expression of TRPM8 was downregulated, while TNFα expression increased significantly (Supplementary Fig. S4). TRPA1 expression was not altered. When *Trpa1* siRNA was introduced into the cells, TRPA1 expression was significantly downregulated; the expression of TNFα was also downregulated at this time (Supplementary Fig. S4). In cold conditions, *Trpm8* siRNA dramatically knocked down TRPM8 expression, and TNFα and NFκB were liberated as shown previously (Supplementary Fig. S5). These results indicated that TRPM8 could directly downregulate TNFα.

**Figure 3:**
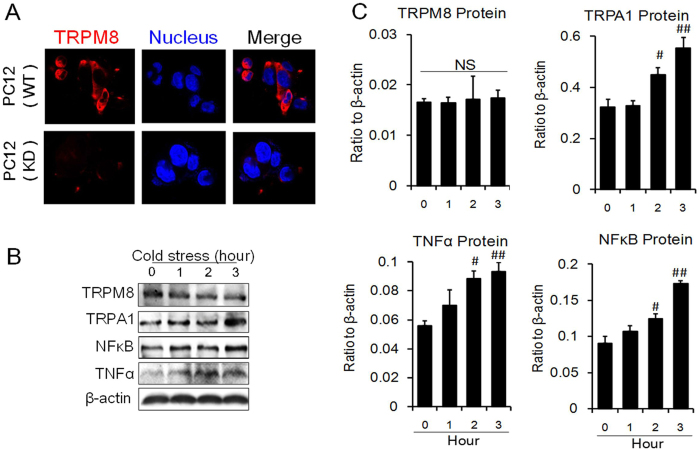
Expression of TRPM8, TRPA1, NFκB and TNFα in PC12 cells with Trpm8 knockdown under cold conditions (4 °C). (**A**) Construction of a Trpm8 (Dylight 649) knockdown stable cell line. WT represents wild type cells. KD signifies the Trpm8 knockdown cells. (**B,C**) Protein expression levels of TRPM8, TRPA1, NFκB and TNFα. NS: no significance. Data are shown as the mean ± S.D. from three experiments. ^#^*P* < 0.05; ^##^*P* < 0.01, v.s. the control (zero time).

To acquire a more thorough understanding of the negative correlation between TRPM8 and TNFα, we constructed a Trpm8 knockdown stable cell line (TRPM8-KD cells) using lentviral vectors. As shown in Fig. 3A, the expression of TRPM8 was notably reduced after transfection with lentivirus containing Trpm8 shRNA. As a result, the intracellular expression of TRPM8 was significantly downregulated. When placed at 4 °C, TRPA1, TNFα and NFκB expression were stimulated at both the transcript and protein level, with little change observed for TRPM8 expression (Fig. 3B and C), implying that Trpm8 knockdown eradicates the suppression of TNFα. The results corresponded to studies using *Trpm8* siRNA and further confirmed the direct effect of cold stress on TRPM8 and its negative correlation with NFκB (Supplementary Fig. S5).

### Co-localization of TRPM8 and NFκB in PC12 cells

**Figure 4:**
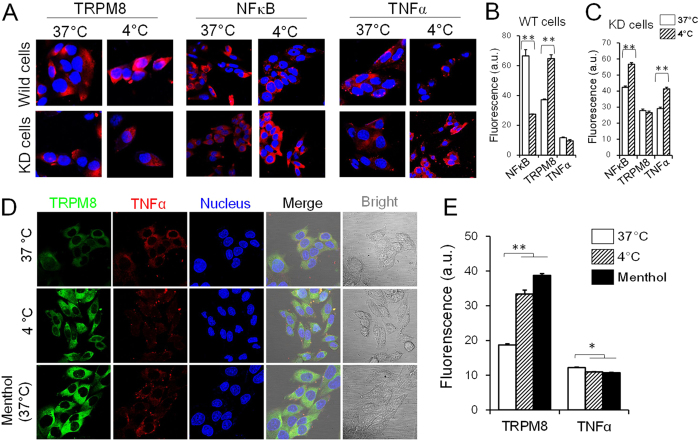
Confocal imaging of TRPM8, NFκB and TNFα expression in PC12 cells. (**A**) The expression of TRPM8, NFκB and TNFα in wild type cells and Trpm8 knockdown cells at 37 °C and 4 °C. KD signifies Trpm8 knockdown cells. (**B**) In wild type cells, TRPM8 was upregulated and NFκB and TNFα were downregulated under cold conditions. (**C**) In KD cells, TRPM8 showed weak expression and NFκB and TNFα expression levels were increased. (**D**) Co-localization of TRPM8 and TNFα in the cytoplasm (cold condition and 500 nM menthol). Data are shown as the mean ± S.D. from three experiments. *P < 0.05; **P < 0.01.

To determine the intrinsic link between TRPM8, NFκB and TNFα, the co-localized expressions of TRPM8 and NFκB were assessed by confocal laser scanning microscopy. As shown in Fig. 4A, both TRPM8 and NFκB were localized in the cytoplasm. Moreover, cold stress promoted TRPM8 expression and suppressed NFκB expression (Fig. 4B). After Trpm8 knockdown, this negative correlation disappeared (Fig. 4C). To further confirm this relationship, we performed the same co-localization experiments. In the same cells, we observed that TRPM8 (shown in green) and TNFα (shown in red) localized to the same sites, suggesting that the two have a spatial relationship. At 4 °C, TRPM8 expression was significantly upregulated and TNFα was significantly downregulated (Fig. 4D and E) and . Menthol, an agonist of TRPM8, can activate both TRPM8 mRNA and protein expression, while downregulating TNFα, suggesting a negative relationship between TRPM8 and TNFα as shown in previous results (Fig. 4D and E; Supplementary Fig. S6).

### Presence of both TRPM8 and NFκB in the cytoplasm leads to a decrease in TNFα expression

**Figure 5:**
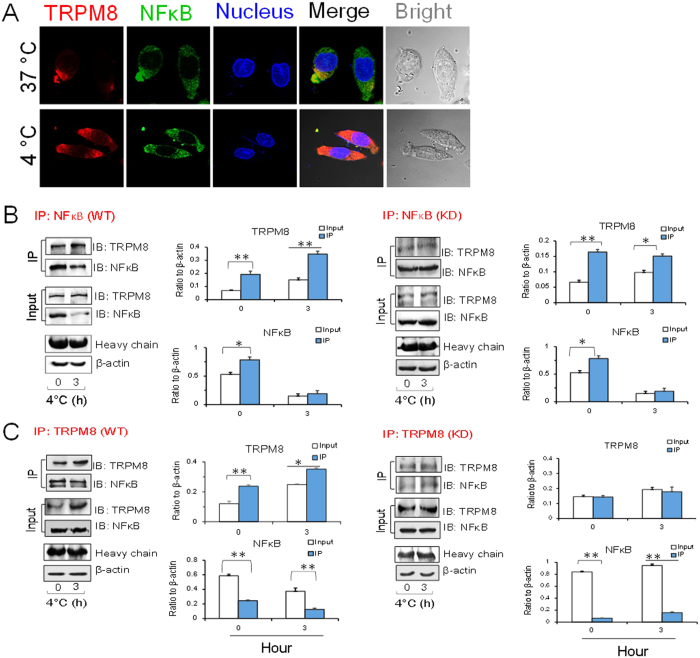
Co-localization of TRPM8 and NFκB in PC12 cells under cold conditions. (**A**) Immunofluorescence assay image of TRPM8 and NFκB in the cytoplasm. WT represents wild type cells. KD represents Trpm8 knockdown cells. (**B**) The results of co-immunoprecipitation (CoIP) of endogenous TRPM8 and NFκB using NFκB antibodies. Western blot analysis was carried out to detect TRPM8 and NFκB. (**C**) Reverse CoIP confirmed interaction between NFκB and TRPM8. CoIPs were also performed with TRPM8 antibodies. Western blot analysis was carried out by TRPM8 and NFκB. Data are shown as the mean ± S.D. from three experiments. *P < 0.05; **P < 0.01.

The above results showed the negative regulation of TNFα by TRPM8. Co-immunofluorescence staining assays suggested that TRPM8 and NFκB co-localized in PC12 cells. To determine whether NFκB mediates TRPM8 regulation of TNFα, co-immunoprecipitation assays were employed to study the interaction between TRPM8 and NFκB. The results showed that TRPM8 and NFκB co-localized and that this interaction was strengthened under cold conditions (Fig. 5A). On the contrary, the combination of TRPM8 and NFκB was reduced in Trpm8 knockdown cells after exposure to cold (Fig. 5B and C), indicating that the inhibitory effect of the cold temperature on NFκB was related to the activation of TRPM8 and that cold-induced TRPM8 stimulation promoted the interaction between TRPM8 and NFκB. This interaction then impeded the effects of NFκB on downstream effectors.

**Figure 6:**
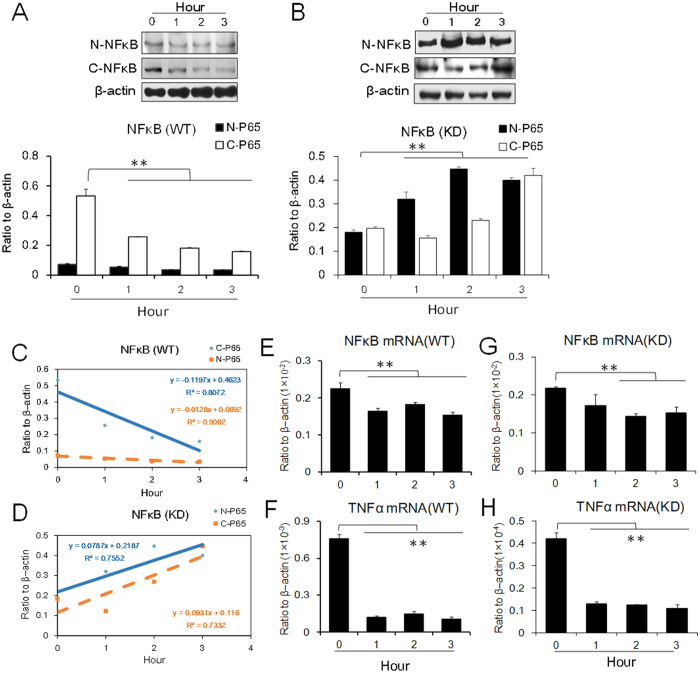
Protein expression of NFκB in PC12 cells under cold conditions (4 °C). (**A,B**) NFκB in the cytoplasm and in the nucleus. (**A**) WT represents wild type cells. (**B**) KD represents *Trpm8* knockdown cells. (**C,D**) Kinetic expression levels of NFκB in both WT and KD cells. C-P65 represents NFκBp65 in the cytoplasm. N-P65 represents NFκBp65 in the nuclei. (**E–H**) The mRNA expression levels of NFκB and TNFα after JSH-23 (8 μM), the inhibitor of NFκB, was applied. (**E**,**F**) WT cells. (**G**,**H**) KD cells. Data are shown as the mean ± S.D. from three experiments. **P < 0.01, *v.s.* the control (zero time).

To further analyze the effect of NFκB on TNFα under cold stress, both nuclear and cytoplasmic extracts were prepared and analyzed by Western blot analysis. Under cold conditions, both nuclear and cytosolic expression of NFκB was downregulated in wild type cells (WT) and upregulated in Trpm8 knockdown cells (KD), suggesting that nuclear localization of NFκB was influenced by cold-induced TRPM8 stimulation (Fig. 6A–D).

Since NFκB is transported into the nucleus to activate transcription of TNFα, the changes in cytoplasmic and nuclear NFκB levels also reflect this regulation. JSH-23 has been reported to have an inhibitory effect on NFκB transcriptional activity^19^. We therefore used JSH-23 to elucidate NFκB and TNFα signaling under cold conditions. The results showed that when pretreated with JSH-23 (8 μM)^20^, the mRNA and protein expression levels of NFκB and TNFα in the WT and KD cells were clearly downregulated after 3 hours of cold exposure, suggesting that the inhibitory effect of cold stress on TNFα was mediated by NFκB (Fig. 6E–H).

### Cooling brain arouses TRPM8 upregulation and TNF downregulation after the cerebral ischemia and reperfusion of mice

**Figure 7:**
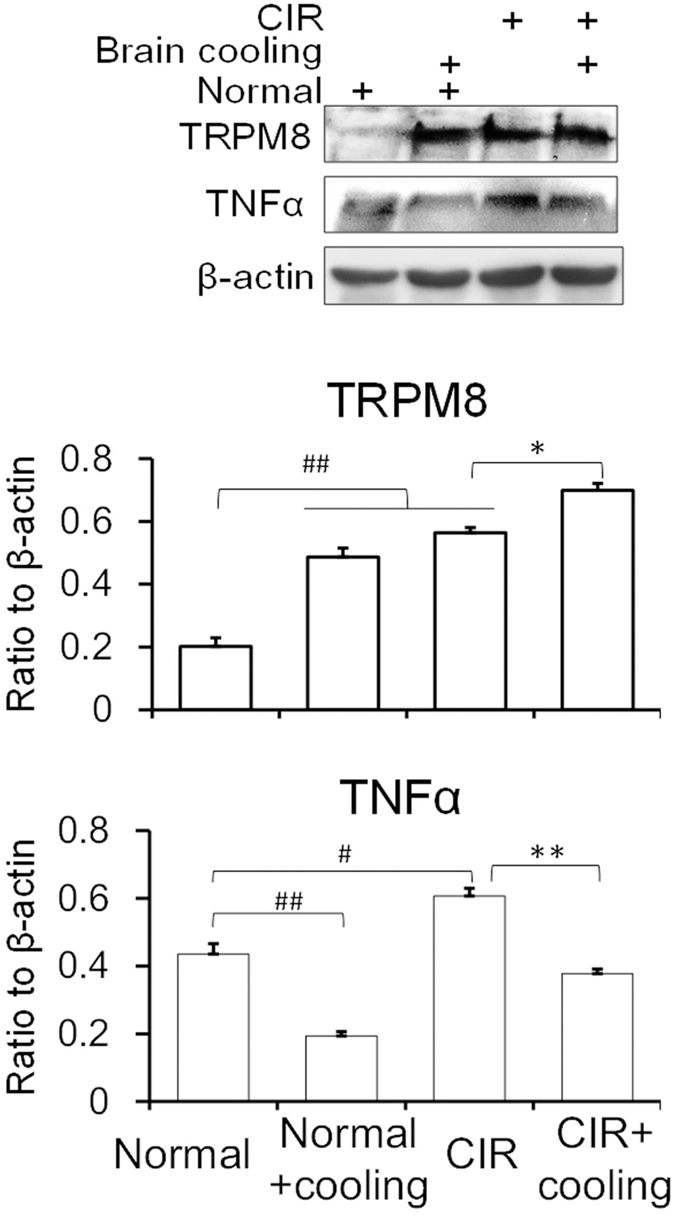
Protein expression of TRPM8 and TNFα in mouse brain with cerebral ischemia-reperfusion (CIR). Brain cooling means putting the anesthetized mouse brain on the artificial ice. Normal means the room temperature (25 °C). Data are shown as the mean ± S.D. from five mice in each group. ^#^*P* < 0.05; ^##^*P* < 0.01, *v.s.* the normal control. *P < 0.05; **P < 0.01, *v.s.* the CIR.

In order to verify the role of cooling therapy in trauma, we conducted the experiments that mouse model with cerebral ischemia reperfusion injury. We put the artificial ice around the brains of anesthesia mice after the cerebral ischemia-operation *in vivo*. The results showed that the expression of TNFα increased distinctly after ischemic injury in the brain of mice although TRPM8 increased in such stress. However, after ischemic brain was cooled for one hour *in vivo*, TNFα was significantly reduced and simultaneously TRPM8 expression was increased further (Fig. 7; Supplementary Fig. S7), which proves TRPM8 had an effect on negative regulation of TNFα, and cold stress played a key role in such regulation.

## Discussion

Our results showed that during cold stress, TRPM8 is highly expressed in the cytoplasm while TNFα expression is downregulated, indicating a negative relationship between TRPM8 and TNFα. Further studies showed that this relationship was mediated by NFκB. In colder temperatures, the affinity between TRPM8 and NFκB was enhanced, thereby inhibiting NFκB’s dissociation with IκB and blocking its nuclear localization, as well as interrupting TNFα transcriptional activation. The present work reflects an interesting phenomenon that cold therapy is capable of attenuating inflammatory injury involved in TRPM8-NFκB-TNFα signaling.

Peripheral nerve cells are involved in cold sensing receptor-mediated induction and excited by nerve impulses. The major receptors involved in this induction process are TRPA1 and TRPM8^21^,^22^. It is generally believed that TRPM8 is more sensitive to low temperatures than TRPA1^23^. The results presented here showed that TRPM8 is more sensitive to a 4 °C environment than a 20 °C environment *in vitro* and it displayed in a temperature-dependent manner. TRPA1 did not have greater sensitivity to low temperatures than TRPM8. When the temperature was set at 34.5 °C in *in vitro* experiment (the mice rectal temperature in cold conditions), TRPM8 of the cells did not response suggesting there are different environments between *in vivo* and *in vitro*. During cold conditions *in vitro*, TNFα was downregulated when TRPM8 expression was increased. Using a siRNA assay, we determined that when TRPM8 was downregulated, TNFα inhibition was reversed despite an upregulation of TRPA1 expression, suggesting that under cold conditions, TRPM8 upregulation reduces the expression of TNFα. The results from Trpm8 knockdown cells are consistent with these findings.

Following transcription, TRPM8 requires the transporter proteins TCAF1 and TCAF2 for transportation to the cell membrane; these proteins are activated upon induction of hypothermia^24^,^25^. We expressed TRPM8 in PC12 cells *in vitro* and detected TRPM8 upregulation under cold conditions, when cells may also express TRPM8. This phenomenon does not necessarily depend on regulation of the neuroendocrine system. Further studies found that at low temperatures, the intracellular TRPM8 levels were significantly increased. We speculate that local temperature changes caused by microcirculation can directly induce upregulation of TRPM8 in neurons of the hypothalamus’ thermoregulatory center and not necessarily through the peripheral nerve cells.

NFκB is a nuclear transcription factor that is mainly activated in response to a variety of gene transcription elements to facilitate the merger of or initiate the transcription of specific genes, including a variety of inflammatory-related factors^26^,^27^. Normally, NFκB and IκBα are localized within the cytoplasm, forming a complex^28^. When IκBα is phosphorylated, NFκB is dissociated from IκBα. NFκBp65, a substrate of NFκB, moves into the nucleus and locates a response element in the corresponding genes, initiating transcription^29^. Thus, suppressing NFκB dissociation from IκBα and interfering with NFκB nuclear translocation can effectively prevent transcription of NFκB’s downstream genes^30^. Using a CoIP assay and confocal microscopy, we confirmed that TRPM8 and NFκB remained localized with one another, suggesting their interaction. NFκB was isolated in the cytoplasm and the nuclei in order to verify the dynamic expressions of NFκB in cold conditions. Furthermore, we found that NFκB in the nucleus decreased while NFκB in the cytoplasm increased, indicating that NFκB remained in the cytoplasm because TRPM8 suppressed its nuclear localization, ultimately inhibiting TNFα gene transcription. Therefore, the increased expression of TRPM8 under cold conditions reduced the inflammatory damage, including necroptosis, mediated by TNFα^31^,^32^.

TRPM8 can be activated by menthol, a natural small molecule^33^. As the intracellular calcium concentration increases, colder temperatures help conduct impulses^34^, allowing the body to adapt to the cold^35^. The present study showed that menthol could activate the TRPM8 expression while simultaneously downregulating the expression of TNFα. This supports the conclusion that activated TRPM8 could downregulate TNFα as shown previously in cold stress studies.

We also carried out studies on TRPM8’s relationship with membrane receptors. There have been a few reports on TRPM8 acting as a signal transducer, as is the case with ion channel TRPM8-RACK1-mediated HIF-1α in prostate cancer cells^36^ and TRPM8 activating the AKT/GSK-3β pathway in breast cancer cells^37^. Here, the results suggested that TRPM8 within intracellular proteins could be used as a signal to mediate downstream signaling to produce a series of biological effects. For example, our findings showed that under cold stress, TRPM8 inhibited the inflammatory response via NFκB-TNFα. These results provided a new perspective on TRPM8, and thus we went on to explore the biological effects of TRPM8 on ion channel membrane receptors.

Cooling has been shown to be an effective means of reducing neuronal damages in clinical settings. Hypothermia plays a critical role in neuroprotective responses that protect the brain from damage^38^, including reduced leukocyte infiltration and decreased adhesion molecules and pro-inflammatory cytokines^39^. Based on the literature about the treatment of tissue damage and prevention of an inflammatory response^15^, we concluded that cold-stimulated TRPM8 might mediate the known therapeutic effects of cooling.

To compare with the cold stress, we conducted the experiments under heat stress and CIR (cerebral ischemia and reperfusion)/OGD (oxygen and glucose deprivation) stress. In the experiments of heat stress, HSP70, NFκB and TNFα mRNA expression was upregulated in both mice and PC12 cells under heat conditions, while TRPM8 was inhibited. Previous studies suggested that HSP70, as a member of the heat shock protein family, is a crucial factor involved in the heat-induced temperature-increase and inflammation. The suppression of HSP70 could reduce the high temperature and the expression of TNFα distinctly, suggesting HSP70 plays a key role during the heat stress^40^,^41^. In the CIR/OGD stress experiments, we found that both TRPM8 and TNFα were upregulated in the mice brain and PC12 cells, which exhibited no negative correlation. However, the expression of TRPM8 upregulated while the expression of TNFα and NFκB downregulated showing a negative correlation when we put the CIR mice brain surrounded by the ice locally and the cells in cold conditions, which suggests that the cold stress is necessary for the regulation of TRPM8 on TNFα. All these could be concluded that there is a specific mechanism of which TRPM8 and TNFα were negatively correlated under cold conditions as we found in our experiments.

Together, this work presents the report of a relationship between TRPM8 and TNFα, and the role of TRPM8 as a binding protein within a cytoplasmic signaling system during cold conditions. For an in-depth understanding of the function of TRPM8, particularly the physiological effects of cold stress when it provides an important experimental basis, we obtained relevant theoretical support: the brain cooling after cerebral trauma can not only reduce the metabolic rate of neurons in the brain but can also direct the negative regulation of TNFα, thereby reducing injury to neurons. Our work suggests the TRPM8 may be a potential target for the prevention and treatment of inflammation.

## Methods

### Experimental animals

Male ICR mice weighing 20–22 g were purchased from Vital River Laboratories (Beijing, China). The animals were housed in temperature- and humidity-controlled rooms, kept on a 12-hour light/dark cycle and provided with unrestricted amounts of rodent chow and drinkable water. The laboratory animal facility was accredited by the AAALAC (Association for Assessment and Accreditation of Laboratory Animal Care International). All experimental procedures were approved by the IACUC (Institutional Animal Care and Use Committee) of Tsinghua University and carried out in accordance with the People’s Republic of China Legislation Regarding the Use and Care of Laboratory Animals (Approval ID: 15-DLJTRPM).

### Chemicals and materials

Menthol (PubChem CID: 16666) was purchased from Sigma Aldrich of Shanghai (Shanghai, China), and 4-methyl-N1-(3-phenyl-propyl)-benzene-1, 2-diamine (JSH-23) (PubChem CID: 16760588), an NFκB activation inhibitor, was purchased from Selleckchem of Shanghai (Shanghai, China). Fetal calf serum (NBCS), fetal bovine serum (FBS), DMEM and RPMI-1640 medium were purchased from GIBCO (Invitrogen, MA, USA). DNA sequences were synthesized by the Sangon Biotech Company (Beijing, China).

### Experimental procedures *in vivo*

#### Expression of TRPM8 in mouse brains under cold conditions and hot conditions

ICR mice were divided into four groups at random (six mice in each group). The normal group was kept at room temperature (25 °C), while the other three groups were exposed to cold conditions (4 °C) (YC-1 Cold-Incubator, Boyikang Laboratory Instrument Co. Ltd., Beijing, China). Cold conditioning lasted 1, 2 or 3 hours, and rectal temperatures (Tr) was measured every hour using a micro-electronic thermal detector (SN2202 Thermal Detector, Sinan Thermal Instrument, Beijing, China). After cold conditioning, all mice were euthanized, and their brains were removed. The hypothalamus was isolated and stored at −80 °C until further use. Simultaneously, the experiments in hot conditions (40 °C) were referenced as that as in cold conditions.

#### Brain cooling in the cerebral ischemia and reperfusion of mice

According to the literature^42^, mice were taken the operation of the cerebral ischemia and reperfusion by carotid artery ligation. After the reperfusion 6 hours, the artificial ice was fixed around the mouse brains in order to decrease the brain temperature in the cerebral hypothermia experiments *in vivo*. The mice were anesthetized with 10% urethane (intraperitoneal injection) before the cooling. The ice were placed around the mice brains for 1 hour. Then the brain was isolated for protein expressions. Normal control consisted of the room temperature (25 °C) groups and the brain cooling groups without the cerebral ischemic operation.

### Experimental procedures *in vitro*

#### Cell culture

The PC12 and HEK 293 T cell lines were obtained from the Cell Culture Center of Chinese Academy of Medical Science (Beijing, China) and maintained at 37 °C in a humidified incubator (Sanyong, Japan) containing 5% CO_2_.

#### Cold and heat conditioning-induced responses in PC12 cells

PC12 cells were maintained at 4 °C and 40 °C for 0, 1, 2 or 3 hours respectively. PC12 cells were then collected, and protein and RNA samples were extracted for mRNA and protein determination.

#### PC12 cells for oxygen-glucose deprivation (OGD)

The OGD insult followed by reperfusion is aimed to mimic the pathological conditions of ischemia *in vitro*. In the OGD model, the normal culture medium was replaced by glucose-free Earle’s solution for 2 hours under humidified atmosphere of 95% N_2_ and 5% CO_2_ at 37 °C and the normal medium was replaced for another 4 hours under normal condition as reperfusion. The control group was maintained under normal conditions with normal culture medium. Cells were collected for mRNA determination^18^.

#### Construction of Trpm8 knockdown stable cell lines using lentiviral vectors

The lentiviral vectors PLL3.7, PMD2.G, pMDLg-pRRE and pRSV-Rev were provided by Dr. Ya-Dong Hu (School of Life Science, Tsinghua University). A multiple cloning site was engineered and placed immediately after the U6 promoter. Hpa I and Xho I were used as restriction sites for cloning. The shRNA sequence was designed by Invitrogen Corporation and produced by Sangon Biotechnology Ltd (Shanghai, China). Oligo sequences were as follows: sense: 5′-TGGGCCATTCTTCAGAACAAGATTCAAGAGATCTTGTTCTGAAGAATGGCCCTTTTTTC-3′; antisense: 5′-TCGAGAAAAAAGGGCCATTCTTCAGAACAAGATCTCTTGAATCTTGTTCTGAAGAATGGCCCA-3′. The lentiviral stock was produced by co-transfecting the optimized packaging plasmid mix (PMD2.G, pMDLg-pRRE and pRSV-Rev) and the transfer vector (PLL3.7) containing the shRNA of TRPM8 into HEK 293 T cells. PC12 cells were then transfected with the packaged lentivirus. Stable PC12 cells with TRPM8 knocked down were produced by sorting with a BD FACSAriaII (BD Medical Biotechnology, New Jersey, USA) according to GFP expression.

#### Immunocolocalization of TRPM8 and NFκB in PC12 cells

Immunohistochemical staining of fixed PC12 cells was performed using anti-TRPM8 antibody (Novus Biological, USA) and anti-NFκB antibody (Santa Cruz, USA). TRPM8 labeling was detected by DyLight 649 (red). NFκB labeling was detected by goat-anti-mouse-FITC (shown in green). Cellular nuclei were stained with DAPI (shown in blue).

#### Co-Immunoprecipitation (CoIP) assays

Total protein was extracted from PC12 cells using a total protein extraction kit (Millipore, Billerica, MA, USA). The cell lysates were incubated with the indicated antibodies at 4 °C for 1–24 h. The immune complex was precipitated with protein G Plus-Agarose (Santa Cruz Biotechnology, Inc., Santa Cruz, USA) for 1 h at 4 °C, washed extensively with a lysis buffer containing NP-40, resolved through a 4–20% gradient SDS-PAGE gel and then analyzed by Western blot analysis.

#### Preparation of nuclear and cytosolic extracts

The nuclear and cytosolic extracts of PC12 cells were prepared using NE-PER nuclear and cytoplasmic extraction reagents (Thermo Scientific) and detected by Western blot assays.

#### Gene silencing by small interfering RNA (siRNA)

For siRNA experiments, siRNA sequences were designed and synthesized by Sigma Aldrich (S2). siRNA was transfected into PC12 cells using RNA Transfectin (TianGen, China) (Rat TRPM8 siRNA ID: SASI_Rn01_00049377; Rat TRPA1 siRNA ID: SASI_Rn01_00270436) in accordance with the manufacturer’s instructions. A nonsense sequence was transfected as the negative control.

#### Real-time PCR and Western blot analysis

mRNA and protein determination were performed using real-time PCR (quality PCR, qPCR)^43^ and Western blot analysis, respectively, as described previously^44^,^45^. For real-time PCR, all primer sequences were designed by NCBI GenBank and produced by Sangon Biotechnology Ltd. (Shanghai, China). The primers are shown in Supplementary Tables S1 and S2. For Western blot analysis, a primary antibody against TRPM8 (NBP1-97311, 127 kDa) was purchased from Novus Biologicals (Littleton, USA), TRPA1 (ab68847, 127 kDa), TNFα (ab199013, 17 kDa) antibodies were purchased from Abcam Trading (Shanghai) Company Ltd. (Shanghai, China) and NFκB (SC-8008, 65 kDa) and β-actin (SC-47778, 43 kDa) antibodies were purchased from Santa Cruz Biotechnology Inc. (Texas, USA). Secondary antibodies goat anti-mouse IgG-HRP (SC-2005) and goat anti-rabbit (SC-2004) IgG-HRP were purchased from Santa Cruz Biotechnology Inc. (Texas, USA). The targeted proteins were visualized with the Super Signal West Femto Chemiluminescent Substrate (Prod# 10095983, Thermo-Scientific, Illinois, USA), and the intensity of the visualized bands was analyzed using QuantityOne software (Bio-Rad). β-actin was used as an internal control. Data are expressed relative to β-actin levels.

#### Calcium imaging

Intracellular Ca^2+^ concentrations were measured using the calcium indicator Fluo-3-AM (S1056; Beyotime, Haimen, China)^46^. Cultured PC12 cells were loaded with 5 μM Fluo-3-AM for 45 min. The cells were then placed at 4 °C as the test group, while the control group was maintained at 37 °C. Using a laser scanning confocal microscopy (LSCM, Zeiss LSM710, Germany), the cells were excited at 488 nm. Ca^2+^-insensitive fluorescence was recorded and calculated using Zen software (Zeiss, Germany).

#### Data analysis

Data are expressed as the mean ± S.D. The data were analyzed using one-way analysis of variance (ANOVA) with F value determination. The F test was carried out using Excel software for Office 2013 (Microsoft, USA). Student’s *t*-test between two groups was performed after the F test. *P* values below 0.05 were considered statistically significant.

## Additional Information

**How to cite this article:** Wang, X.-P. *et al*. TRPM8 in the negative regulation of TNFα expression during cold stress. *Sci. Rep.* **7**, 45155; doi: 10.1038/srep45155 (2017).

**Publisher's note:** Springer Nature remains neutral with regard to jurisdictional claims in published maps and institutional affiliations.

## Supplementary Material

Supplementary Information
