# Supplementary material for: TRPM8 in the negative regulation of TNFα expression during cold stress
Source: Sci Rep. 2017 Mar 23;7:45155. doi: 10.1038/srep45155 (PMC5362914; doi:10.1038/srep45155)
Supplement: Supplementary Information [file srep45155-s1.pdf]

TRPM8 in the negative regulation of TNFα expression during cold stress

Xin-Pei Wang[1†], Xuan Yu[1†], Xiao-Jin Yan[1], Fan Lei[2], Yu-Shuang Chai[1], Jing-Fei Jiang[1], Zhi-Yi Yuan[1], Dong-Ming Xing[1], Li-Jun Du[1*]

[1]MOE Key Laboratory of Protein Sciences, Laboratory of Molecular Pharmacology and Pharmaceutical Sciences, School of Life Sciences, Tsinghua University, Beijing 100084, China

[2]School of Pharmacology and Pharmaceutical Sciences, Tsinghua University, Beijing 100084, China

[†] **Equal in contribution**

[*] **Correspondence should be addressed to**

MOE Key Laboratory of Protein Sciences,

Laboratory of Molecular Pharmacology and Pharmaceutical Sciences

School of Life Sciences

Tsinghua University,

Beijing 100084, China

Tel: +86-10-62796270

Email: lijundu@mail.tsinghua.edu.cn

Supplementary information includes:

Supplementary Figure S1-S11, Table S1 and S2.

Supplementary information
Figure S1

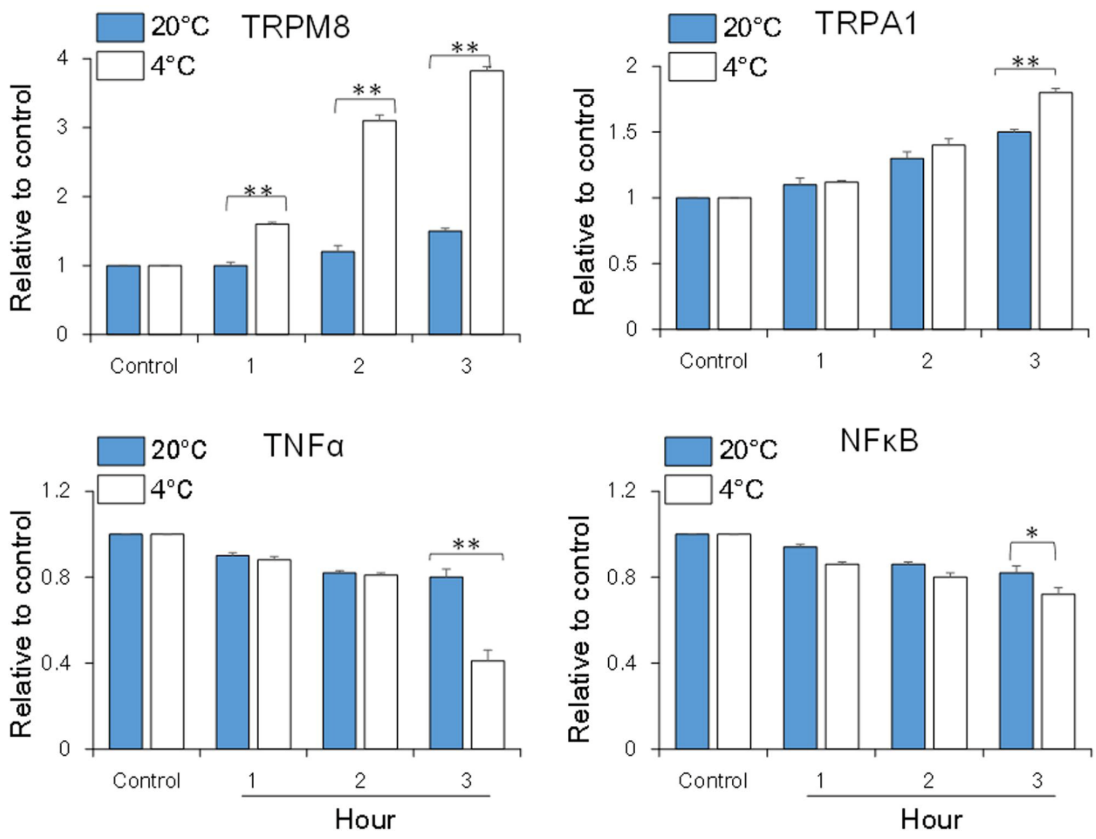

Figure S1. Comparison of the mRNA expressions of TRPM8, TRPA1, TNFα and NFκB of PC12 cells in between 20 °C and 4 °C conditions. Data are shown as the mean ± S.D. from three independent experiments. *, $P < 0.05$; **, $P < 0.01$.

Figure S2

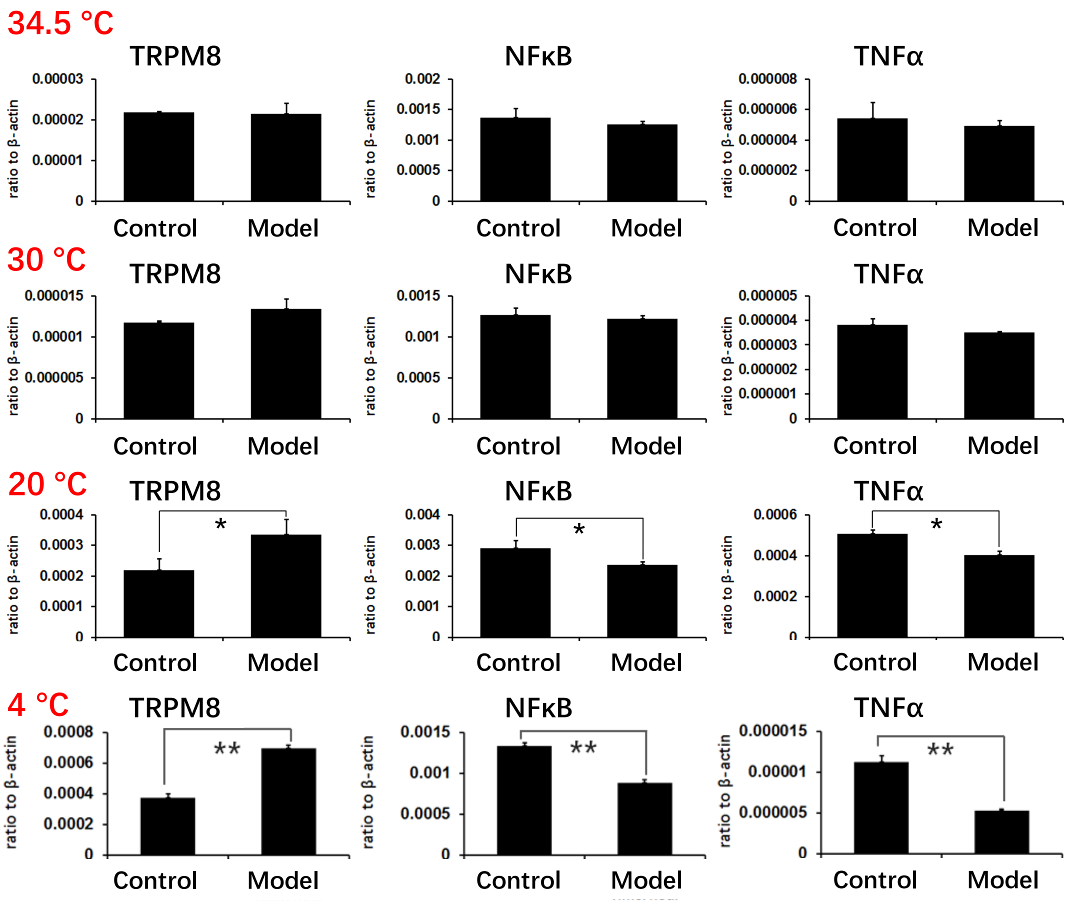

Figure S2. The mRNA expressions of TRPM8, NFκB and TNFα in PC12 cells under different temperature *in vitro*. Control: 37 °C. Model: Cold conditions. Data are shown as the mean ± S.D. from three independent experiments. *, $P < 0.05$; **, $P < 0.01$.

Figure S3

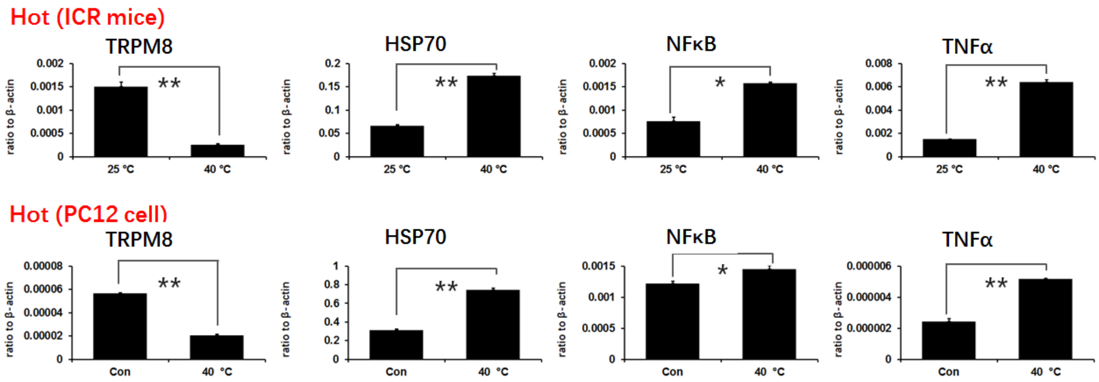

Figure S3. The mRNA expressions of TRPM8, HSP70, NFκB and TNFα in heat stress (40 °C) both *in vivo* and *in vitro*. Data are shown as the mean ± S.D. from five mice in each group *in vivo* and three independent experiments *in vitro*. *, $P < 0.05$; **, $P < 0.01$.

Figure S4

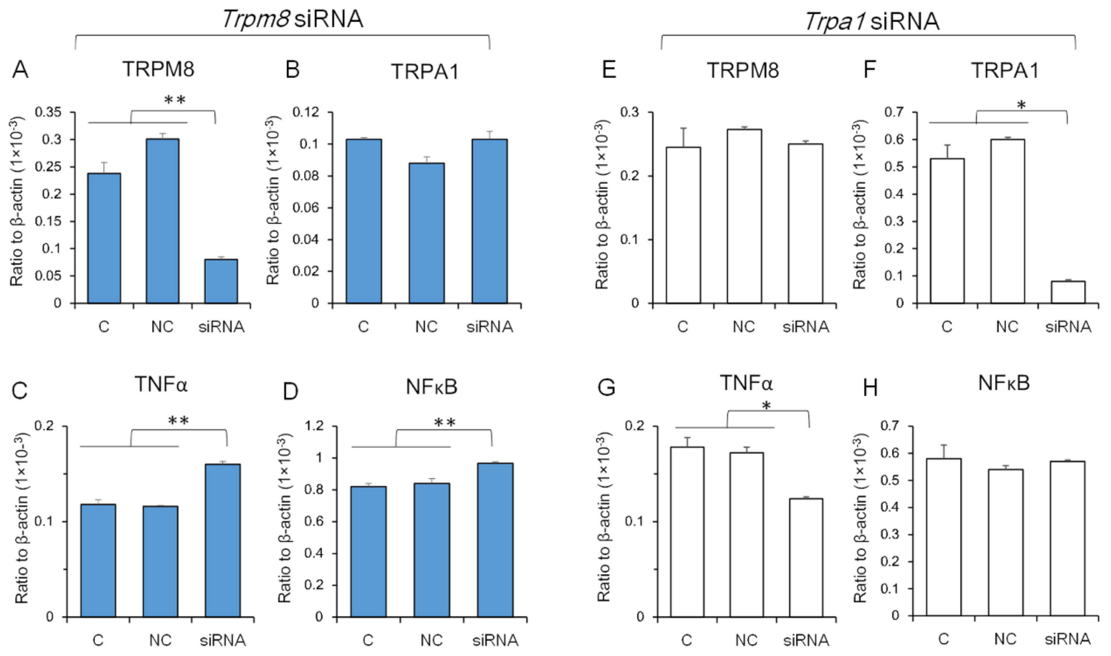

Figure S4. Effect of *Trpm8*siRNA and *Trpa1*siRNA (siRNA) on the mRNA expressions of TRPM8, TRPA1, TNFα and NFκB in PC12 cells in normal conditions (37 °C). (A) - (D): *Trpm8*siRNA. (E) - (H): *Trpa1*siRNA. Data are shown as the mean ± S.D. from three independent experiments. *, $P < 0.05$; **, $P < 0.01$.

Figure S5

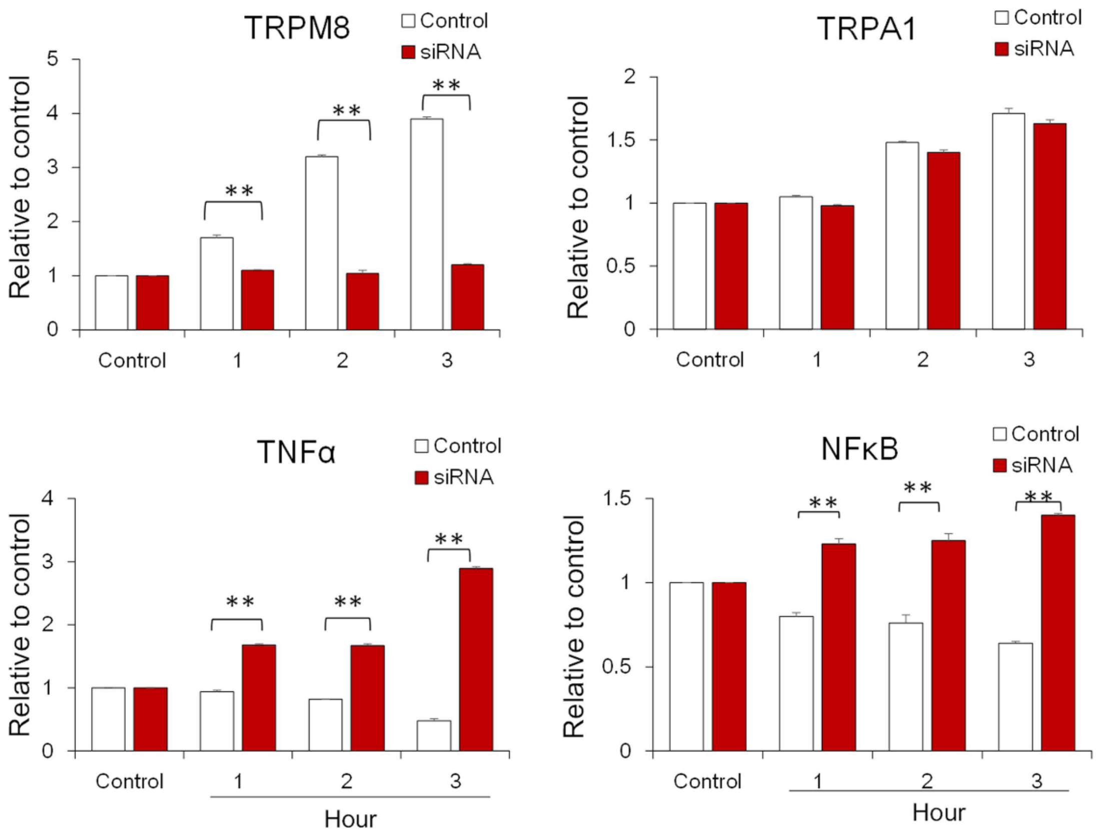

Figure S5. Effect of *Trpm8*siRNA (siRNA) on the mRNA expressions of TRPM8, TRPA1, TNFα and NFκB in PC12 cells in cold conditions (4 °C). Data are shown as the mean ± S.D. from three independent experiments. **, $P < 0.01$.

Figure S6

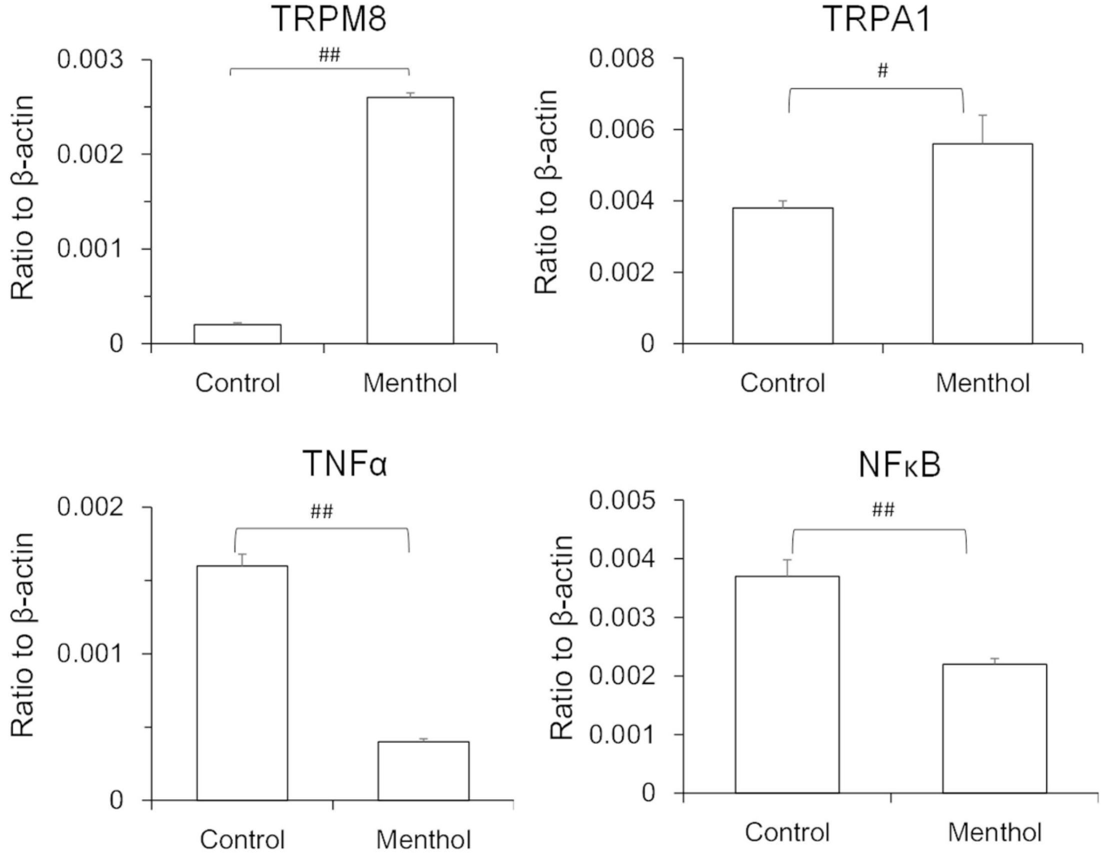

Figure S6. Effect of menthol on the mRNA expressions of TRPM8, TRPA1, TNFα and NFκB in PC12 cells. Data are shown as the mean ± S.D. from three independent experiments. #, $P < 0.05$; ##, $P < 0.01$.

Figure S7

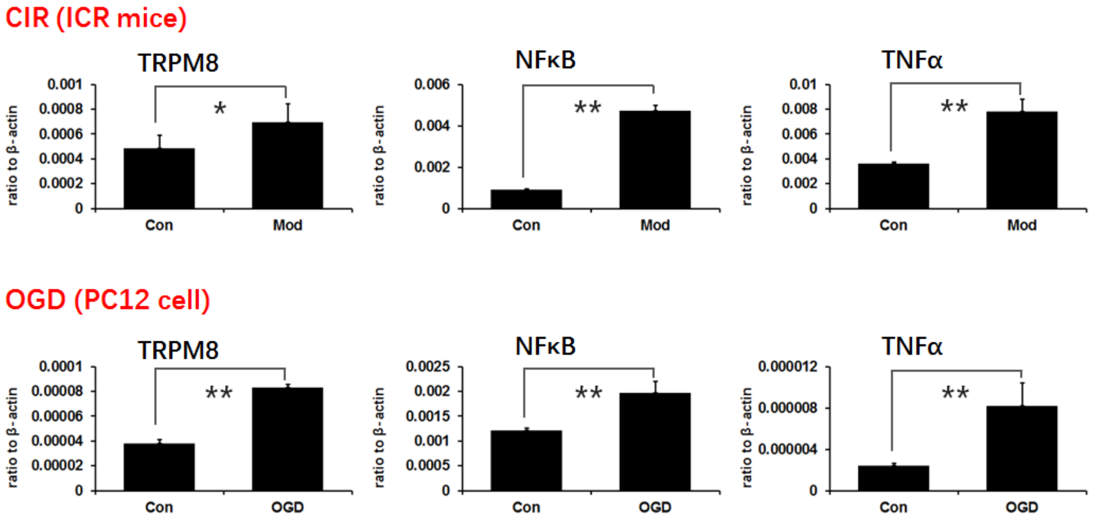

Figure S7. The mRNA expressions of TRPM8, NFκB and TNFα in ischemia and reperfusion stress under normal conditions (*in vivo*: room temperature, *in vitro*: 37 °C). Con represents control, Mod means model. CIR: cerebral ischemia and reperfusion, OGD: oxygen and glucose deprivation. Data are shown as the mean ± S.D. from five mice in each group *in vivo* and three independent experiments *in vitro*. *, $P < 0.05$; **, $P < 0.01$.

Figure S8

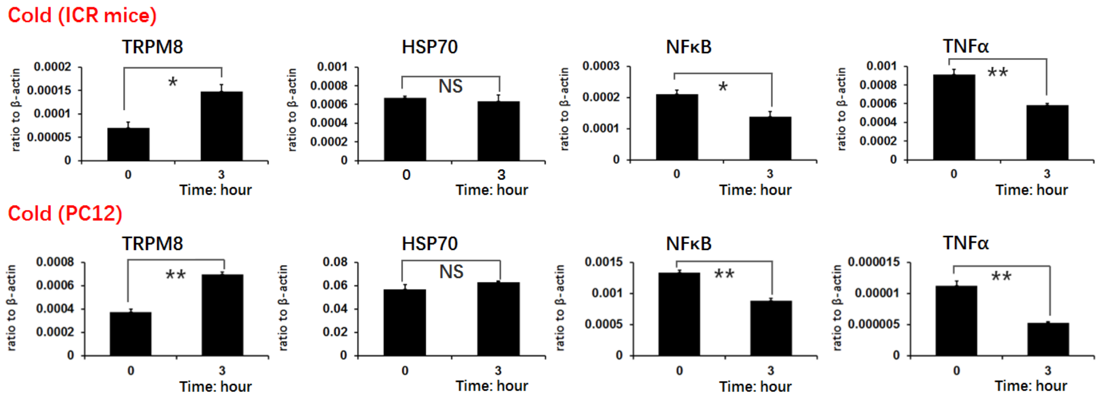

Figure S8. The mRNA expressions of TRPM8, HSP70, NFκB and TNFα under cold stress (4 °C) both *in vivo* and *in vitro*. Data are shown as the mean ± S.D. from five mice in each group in vivo and three independent experiments *in vitro*. *, $P < 0.05$; **, $P < 0.01$.

Figure S9

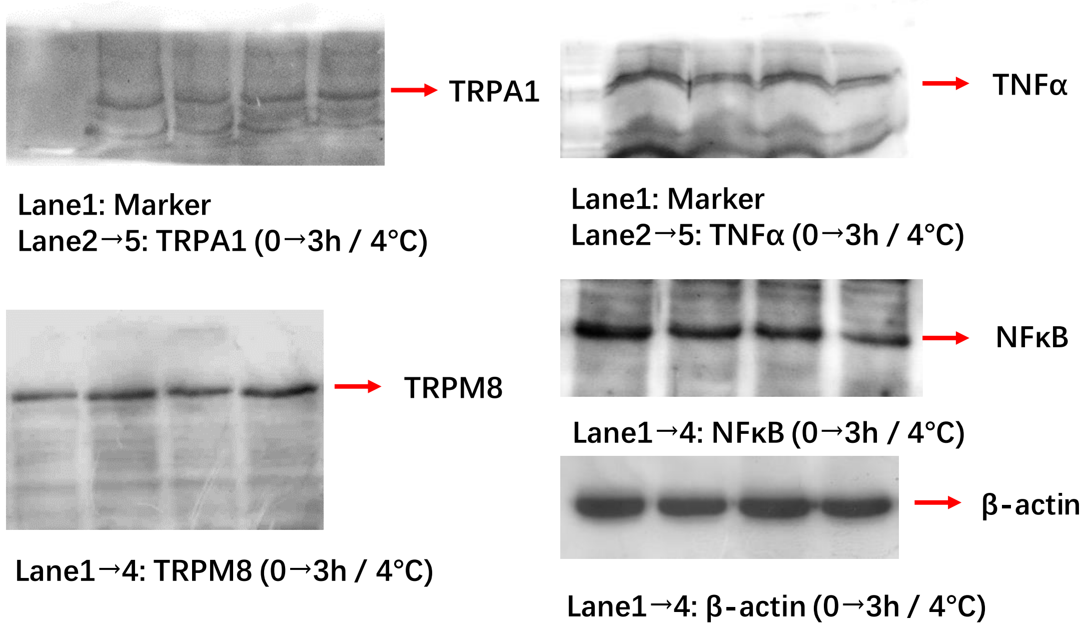

Figure S9. Original blots of cropped western blots of Figure 1. The expression levels of TRPM8, TRPA1, NFκB and TNFα in mouse brains under cold conditions.

Figure S10

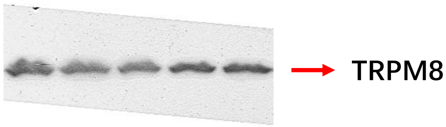

Lane1: Positive control
Lane2→5: TRPM8 (0→3h / 4°C)

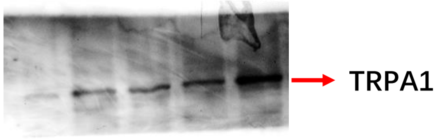

Lane1: Positive control
Lane2→5: TRPA1 (0→3h / 4°C)

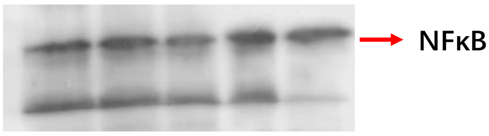

Lane1→4: NFκB (0→3h / 4°C)
Lane5: Positive control

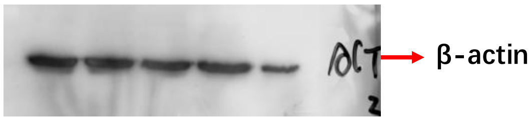

Lane1→4: β-actin (0→3h / 4°C)
Lane5: Positive control

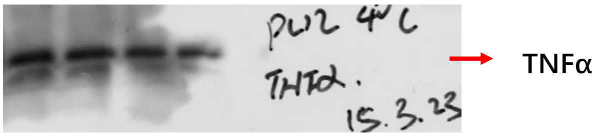

Lane1→4: TNFα (0→3h / 4°C)

Figure S10. Original blots of cropped western blots of Figure 2. Expression of TRPM8, TRPA1, NFκB and TNFα in PC12 cells under cold conditions.

Figure S11

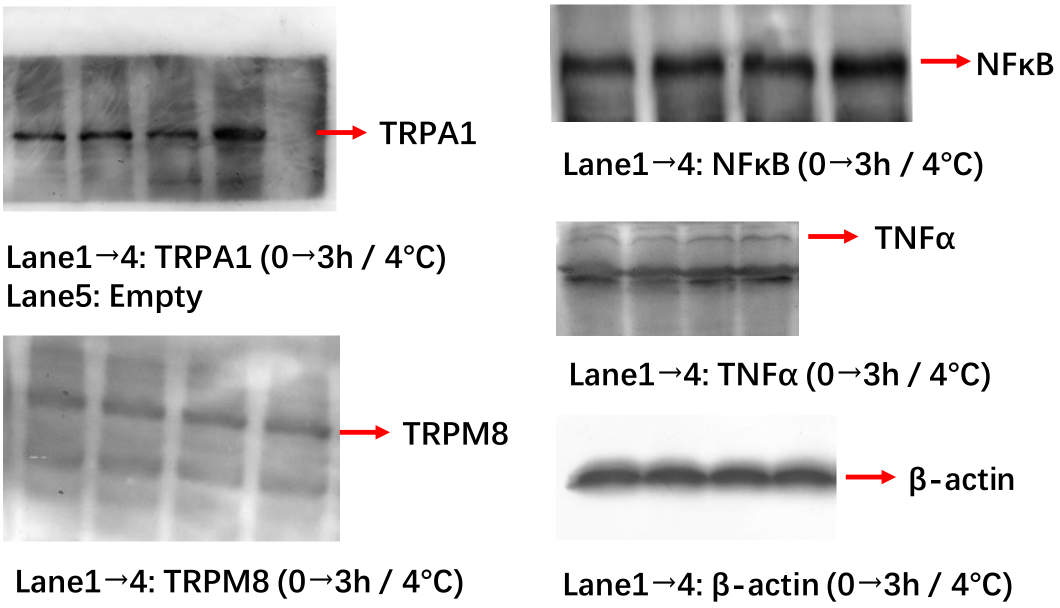

Figure S11. Original blots of cropped western blots of Figure 3. Expression of TRPM8, TRPA1, NFκB and TNFα in PC12 cells with Trpm8 knockdown under cold conditions.

Figure S12

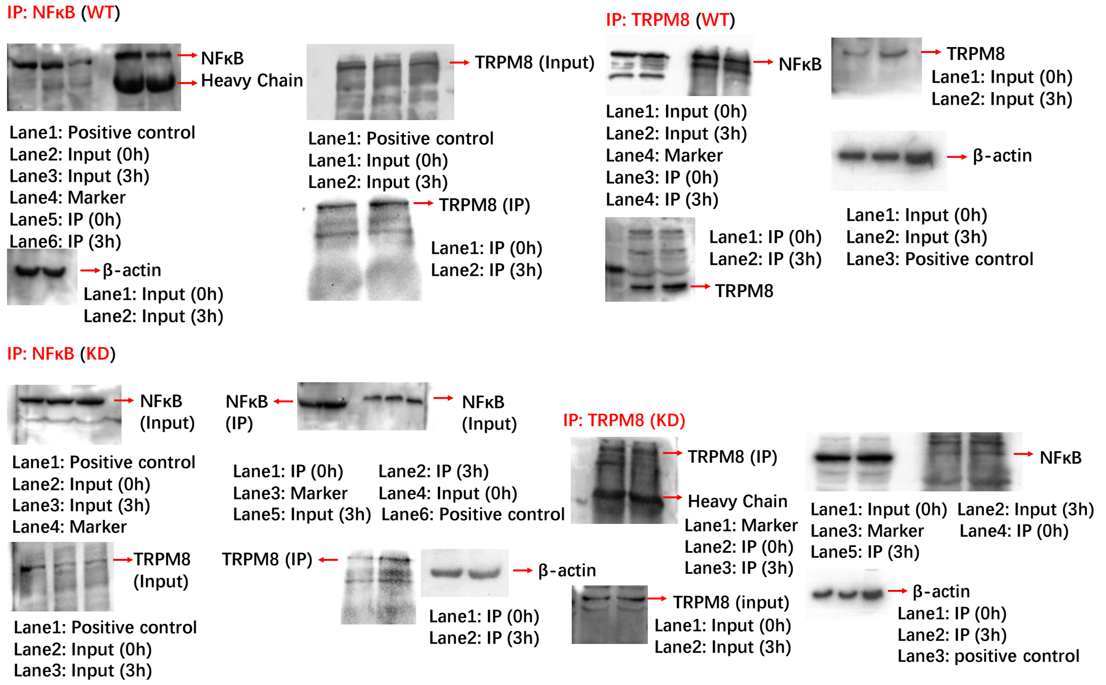

Figure S12. Original blots of cropped western blots of Figure 5. Co-immunoprecipitation (CoIP) of endogenous TRPM8 and NFκB using NFκB antibodies and reverse CoIP performed with TRPM8 antibodies in PC12 (WT) and (KD) respectively.

Figure S13

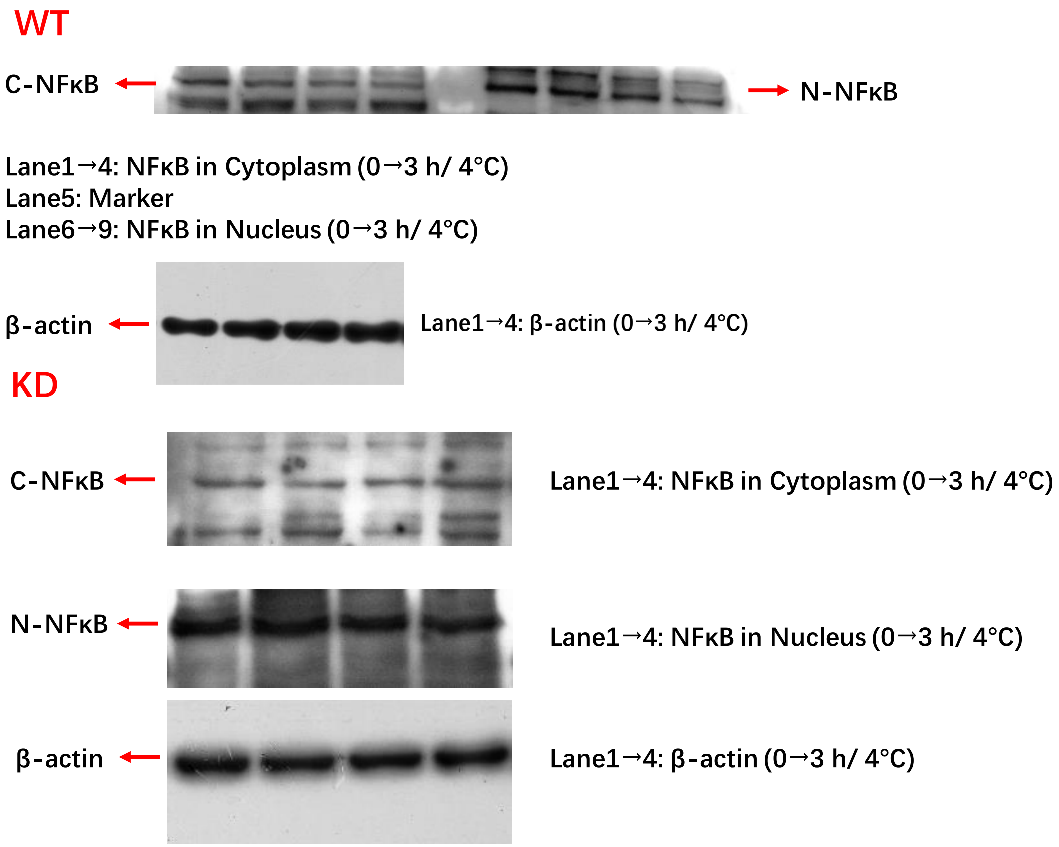

Figure S13. Original blots of cropped western blots of Figure 6. Protein expression of NFκB in PC12 cells under cold conditions (4 ºC).

Figure S14

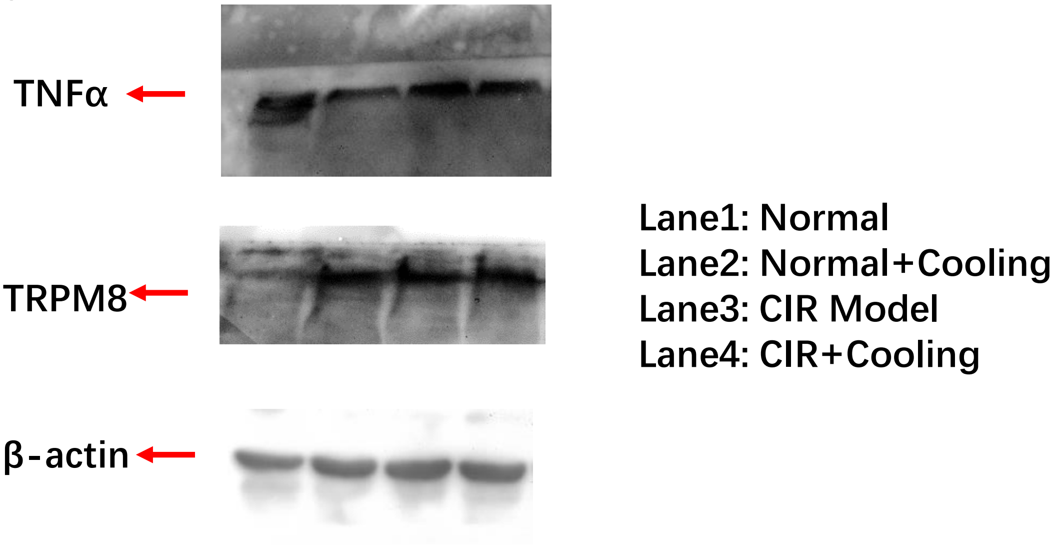

Figure S14. Original blots of cropped western blots of Figure 7. Protein expression of TRPM8 and TNFα in mouse brain with cerebral ischemia-reperfusion (CIR)..

Table S1

Table S1 Primer for mouse

Gene	Sense	Anti-sense
TRPM8	TATGAGACCCGAGCAGTGGA	CAGGCTGAGCGATGAAATGC
TRPA1	GTCCAGGGCGTTGTCTATCG	CGTGATGCAGAGGACAGAGAT
NFκB	GGAGGCATGTTCGGTAGTGG	CCCTGCGTTGGATTTCGTG
TNFα	CCCTCACACTCAGATCATCTTCT	GCTACGACGTGGGCTACAG
β-actin	AGGCCACACAAATAGGGTCC	TTGTGGACACTGCCCCATTC

Table S2

Table S2 Primer for PC12 cells (rat)

Gene	Sense	Anti-sense
TRPM8	TCATACCCACCTGCTGCTTG	CCTGGGCAAAACACACGATG
TRPA1	GCAGCATTTTCAGGTGCCAA	CGCTGTCCAGGCACATCTTA
NFκB	CGCCTGAGACCCGAGACAAG	CTGCCTCCTGCTCCACTGAC
TNFα	CTCCAGCTGGAAGACTCCTCCCAG	CCCGACTACGTGCTCCTCACC
β-actin	CCGTAAAGACCTCTATGCCAACA	CGGACTCATCGTACTCCTGCTT
